# A Case Report of Patent Urachus With Perinatal Cardiac Abnormality

**DOI:** 10.1002/ccr3.70626

**Published:** 2025-07-08

**Authors:** Hanan Mohammed Alzahrani

**Affiliations:** ^1^ Department of Diagnostic Radiology College of Applied Medical Sciences, Taibah University Medina Saudi Arabia

**Keywords:** case report, congenital, cyst, newborn, patent urachus, umbilical problems

## Abstract

Patent urachus is a rare urachal anomaly. This case involves a newborn female with a cyst near the umbilicus and a small patent ductus arteriosus with left‐to‐right shunting. A low‐dose fluoroscopic cystogram revealed contrast entering a tubular structure extending from the bladder to the umbilicus, with evidence of leakage at the umbilical site, confirming a patent urachus. Surgical treatment was performed through an infraumbilical incision to remove the persistent urachus up to the bladder. The patient recovered well postoperatively and was discharged in stable condition to her parents' care. Early diagnosis and management are essential for favorable outcomes in such anomalies.

**Summary**

- Given the potential association of urachal anomalies with congenital conditions such as cardiac defects, omphalocele, and bladder exstrophy, this case highlights the importance of comprehensive prenatal and postnatal evaluation to guide timely, multidisciplinary intervention and optimize neonatal outcomes.

## 1 Introduction

Patent urachus is a condition belonging to urachal anomalies, a rare group of disorders that arise from the maldevelopment of embryonic tissue responsible for draining the embryonic bladder [1]. In normal cases, the urogenital sinus is reached by the upper portion of the allantois, while the umbilical cord surrounds the rest of the allantois. This segment forms a fibrous structure known as the urachus, connecting the top of the bladder to the umbilicus [2, 3]. By the 12th gestational week, this fibrous structure is expected to close, transforming into a ligament called the median umbilical ligament. Yet, when this ligament fails to fully form, various urachal anomalies can occur, with the type of anomaly depending on the site and extent of imperfect closure. Neonates with this condition typically suffer from several symptoms, including continuous umbilical urine leakage, inflammation, irritability, a prominent umbilical cord, and a detectable mass in the abdominal area at birth or shortly thereafter [4, 5].

Urachal anomalies are typically identified via antenatal ultrasound and are categorized into four types according to the continued presence of the embryonic urachal residuals: urachal cyst, umbilical‐urachal sinus, vesicourachal diverticulum, and patent urachus [6]. However, the emergence of symptoms or unusual physical findings mentioned earlier, indicative of urachal remnants, should be assessed to diagnose and determine the specific type of urachal anomaly. In many cases, urachal remnants may be associated with umbilical cord cysts and an enlarged umbilical cord, causing a risk of under‐diagnosis or misdiagnosis. A late diagnosis can result in complications like abdominal pain, infection, and the risk of transforming into malignancy [1, 2].

This case report describes a newborn with congenital patent urachus, including the findings, diagnoses, radiologic images, and treatment. This report underscores the rare association of patent urachus with perinatal cardiac abnormality, highlighting the need for early diagnosis, comprehensive evaluation, and multidisciplinary management to optimize neonatal care.

## 2 Case Presentation

### 2.1 Clinical Background and Maternal History

Early identification and surgical management of patent urachus in neonates is crucial to prevent complications, especially when associated with concurrent anomalies such as patent ductus arteriosus. In this case, a preterm female infant, born at 33 weeks of gestation, weighing 1.65 kg, was delivered via emergency caesarean section. The infant was one of a set of spontaneous twins and presented with a patent allantoic well‐defined, irregularly shaped allantoic cyst. The cyst measures approximately 1.4 cm × 0.7 cm with no internal vascularity. The delivery was performed under the care of a 38‐year‐old diabetic mother (gravida 5, para 4) who presented with regular uterine contractions and abdominal pain but no vaginal bleeding. Foetal movements were reported to be within the normal range.

### 2.2 Initial Management and Radiological Confirmation

At birth, the umbilical cord was clamped and cut under ultrasound guidance by the pediatric surgical team. The infant initially presented with respiratory distress syndrome, thrombocytopenia, and neonatal jaundice, all of which were resolved with appropriate medical management. During the neonatal period, the infant developed a urinary tract infection caused by methicillin‐resistant staphylococcus aureus (MRSA), which was successfully treated with a 10‐day course of antibiotics.

On the first day of life, a pediatric echocardiogram revealed a small patent ductus arteriosus (PDA) shunting left to right, measuring 2 mm, and a mildly hypertrophied left ventricle.

**FIGURE 1:**
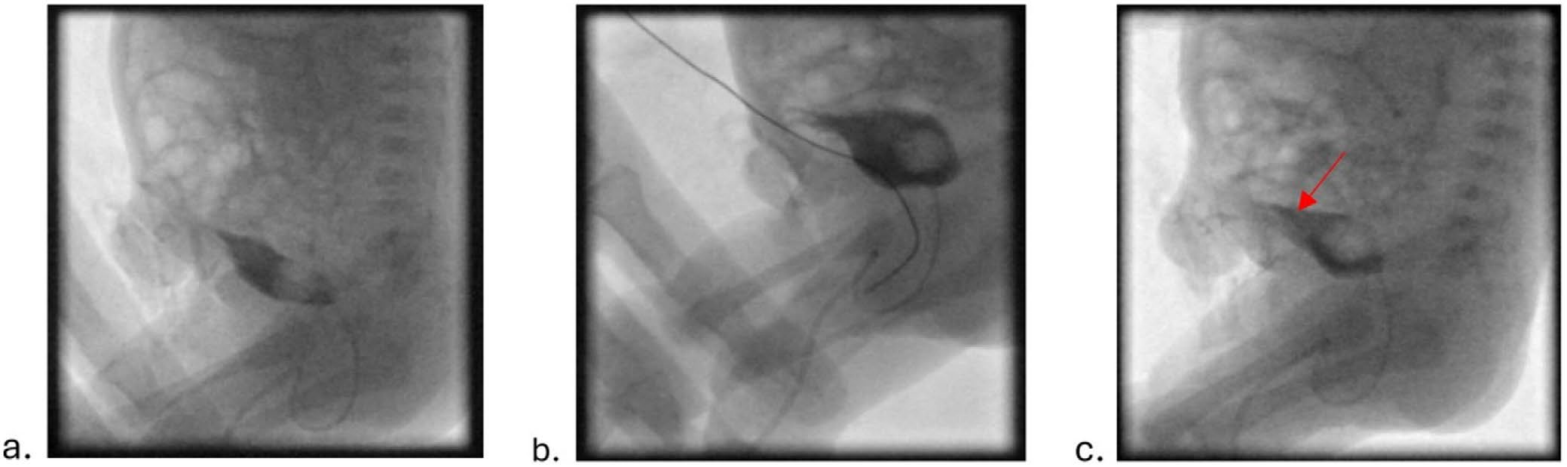
The bladder was filled with contrast, which immediately filled a tubular structure from the dome of the bladder into the base of the remaining umbilical cord. There was free spillage of the contrast media from the umbilicus, consistent with patent urachus (a–c = Lateral views captured during various stages of filling). The red arrow in image (c) pointed to the leakage in bladder.

Low‐dose fluoroscopy was used to perform a cystogram to rule out anomalies and reflux when the patient was 28 days old. The fluoroscopy time was 0:44 min, and 35 mL of Xenetix 300 contrast, diluted with normal saline, was administered via a bladder catheter by gravity. A non‐obstructive bowel gas pattern was noted. The bladder was filled with contrast, which immediately filled a tubular structure extending from the dome of the bladder to the base of the remaining umbilical cord (look at Figure 1). The red arrow in Figure 1c shows that free spillage of contrast from the umbilicus was observed, indicating findings consistent with patent urachus. Radiation safety principles were strictly applied during the fluoroscopic procedure to minimize patient exposure while ensuring diagnostic efficacy.

### 2.3 Treatment Approach and Recovery

The patient underwent surgical treatment. An infraumbilical incision was made to dissect the persistent urachus up to its junction with the bladder. The urachus was then divided, and the bladder was sutured. Hemostasis was assured, and the wound was closed. Postoperative care was provided, and the patient was discharged with her parents.

## 3 Conclusion and Results

At the 1‐month follow‐up, the surgical site had healed well, and the infant remained asymptomatic. Echocardiographic assessment of the patent ductus arteriosus indicated that further follow‐up is required to determine whether surgical intervention will be necessary.

This case underscores the rare occurrence of a patent urachus coexisting with an umbilical cord cyst and PDA in a newborn. It underscores the importance of early, multidisciplinary evaluation for associated anomalies, as well as timely surgical intervention to ensure favorable outcomes. The patient's uneventful recovery and excellent prognosis emphasize the importance of a multidisciplinary approach in managing such cases. Further research is needed to elucidate potential genetic or embryological links between urachal anomalies and other congenital defects.

## 4 Discussion

The urachus is a vestigial structure in the fetus, functioning as a retroperitoneal connection between the fetal bladder and the allantoic cavity within the umbilical cord. It plays a key role in the elimination of fetal waste during development [1]. Normally, the urachus obliterates after birth, forming the median umbilical ligament. However, it may remain patent in rare cases, leading to various clinical abnormalities. Urachal anomalies are often associated with other congenital conditions, particularly involving the umbilical cord, urinary tract, or cardiovascular system [7]. This case highlights the co‐occurrence of a patent urachus, umbilical cord cyst, and small PDA.

A patent urachus results from the partial closure of the urachal lumen during fetal development. While the exact cause remains unclear, potential contributing factors include abnormal embryological processes, genetic predispositions, or intrauterine disruptions [6, 8, 9].

Urachal anomalies are frequently associated with other congenital abnormalities, including bladder exstrophy, omphalocele, prune belly syndrome, and cardiac defects. Fetuses with omphalocele, in particular, are at increased risk of congenital heart disease and perinatal cardiac abnormalities, such as persistent pulmonary hypertension [7, 10] Urachal anomalies have also been reported in syndromic conditions like the VACTERL association. Recognizing these associations supports a multidisciplinary approach to diagnosis and management, ensuring comprehensive neonatal care [11].

In this case, the coexistence of a urachal anomaly with a PDA underscores the need for thorough prenatal and postnatal evaluation. Persistent pulmonary hypertension and cardiac abnormalities require close echocardiographic monitoring. Furthermore, the presence of an umbilical cord cyst, as observed in this patient, may indicate other underlying anomalies and warrants further investigation. Although both urachal and cardiac anomalies are congenital, current evidence does not support a definitive shared embryologic or genetic origin, emphasizing the need for further developmental and genetic studies to explore potential links.

Imaging plays a pivotal role in diagnosing and managing urachal anomalies. Ultrasound is often the preferred modality, offering a non‐invasive method to visualize the patent urachus and associated abnormalities, in addition to cystoscopic and fluoroscopic techniques with contrast media to delineate the umbilical‐vesical tract [6, 8, 9]. Advanced imaging techniques such as CT and MRI can provide high‐resolution details, including fluid, air, or stones in the patent urachus, and assess for complications like infection or malignancy [6]. In this case, fluoroscopy was used to confirm the diagnosis and delineate the umbilical‐vesical tract, while ultrasound guided the removal of the umbilical cord.

The management of a patent urachus typically involves early surgical intervention to prevent complications such as infection, urinary tract obstruction, or, in rare cases, malignant transformation. For asymptomatic cases, close monitoring is essential. In this case, surgical correction was performed using an infraumbilical incision to dissect and excise the urachal remnant up to its bladder junction. The procedure also included the repair of the bladder dome [12].

The patient underwent scheduled surgical correction of the patent urachus with no complications. Although an open infraumbilical approach was used in this case, minimally invasive techniques such as laparoscopic excision of the urachal remnant have been reported as effective alternatives, offering potential benefits including reduced postoperative pain, shorter hospital stays, and improved cosmetic outcomes [12]. Postoperative recovery was uneventful, and the patient was discharged in good health. The PDA is expected to close spontaneously without intervention. Long‐term follow‐up is necessary to monitor for potential complications such as recurrent urinary tract infections, bladder dysfunction, or incomplete resolution of the anomaly. In this case, the patient was discharged with instructions for routine pediatric follow‐ups, including echocardiography to evaluate the spontaneous closure of the PDA.

This report highlights that patent urachus may be associated with perinatal cardiac abnormalities, highlighting the critical need for comprehensive and early evaluation of both the urinary and cardiac systems in affected infants to ensure proper diagnosis and management. It contributes to clinical awareness by educating healthcare providers on recognizing and managing co‐occurring congenital defects, potentially improving patient outcomes. However, the generalizability of the findings is limited due to the unique nature of the case, and the lack of control or comparative data prevents the establishment of broader conclusions. Additionally, the potential for selection bias in choosing the case and the absence of long‐term follow‐up data restrict the ability to assess the full clinical course and outcomes. Therefore, while the report provides important information, its applicability to broader patient populations should be considered with caution.

## Author Contributions

**Hanan Mohammed Alzahrani:** conceptualization, formal analysis, writing – original draft, writing – review and editing.

## Consent

Written informed consent was obtained from the guardian for publication of this case report.

## Conflicts of Interest

The author declares no conflicts of interest.

## Data Availability

The data that support the findings of this study are available from the corresponding author upon reasonable request.
